# Ibuprofen does not have an adverse impact on semen parameters

**DOI:** 10.1007/s10815-018-1330-2

**Published:** 2018-10-17

**Authors:** Parviz K. Kavoussi, Melissa S. Gilkey, Caitlin Hunn, G. Luke Machen, Shu-Hung Chen, J. David Wininger, Keikhosrow M. Kavoussi, Shahryar K. Kavoussi

**Affiliations:** Austin Fertility & Reproductive Medicine/Westlake IVF, 300 Beardsley Lane, Building B, Suite 200, Austin, TX 78746 USA

**Keywords:** Ibuprofen, Semen analysis, Sperm

## Abstract

**Purpose:**

A recent study suggested that ibuprofen may alter testicular physiology in a state of compensated hypogonadism, but only evaluated spermatogenic cells in a laboratory ex-vivo model with no significant effect, and found no significant change in follicle stimulating hormone (FSH) in men treated with ibuprofen. The study did not evaluate the impact of ibuprofen use on clinical semen parameters, which has not been assessed to date. The purpose of this study was to evaluate the impact of ibuprofen on semen parameters.

**Methods:**

In a retrospective chart review from October 2012 to February 2018, 64 men had semen analyses revealing leukocytospermia and were treated with a 3-week course of ibuprofen 600 mg every 8 hours (1800 mg per day) and had a repeat semen analyses 3 weeks later.

**Results:**

Of the 64 men diagnosed with leukocytospermia, 51 returned for post-treatment semen analyses. Parameters included semen volume, sperm concentration, motility, TMC, and forward progression. Morphology was excluded as it could not be standardized between assessments with strict Kruger criteria versus WHO fourth edition criteria depending on the lab in which it was performed. The mean age of these men was 35 (SD 4.6). There was no difference in mean abstinence intervals prior to semen analyses for the pre-treatment and post-treatment data. There was no significant difference in pre-treatment and post-treatment semen volumes, sperm concentrations, motility, TMC, or forward progression.

**Conclusions:**

Among men with leukocytospermia, the treatment with a 3-week course of ibuprofen at 1800 mg per day did not demonstrate a significant adverse impact on semen volume, sperm concentration, motility, TMC, or forward progressive motility when compared to pre-treatment semen analyses parameters.

## Introduction

Approximately 15% of couples attempting to conceive and achieve a pregnancy will have difficulty doing so and are considered subfertile [1–3]. Twenty percent of infertility cases are solely due to male factor, and an additional 40% of infertility cases involve both male and female factors, indicating that in 60% of infertility cases, there is some male factor involvement [3, 4]. A recent study suggested that ibuprofen may alter testicular physiology in a state of compensated hypogonadism, but only evaluated spermatogenic cells in a laboratory ex-vivo model with no significant effect, and found no significant change in follicle stimulating hormone (FSH) in men treated with ibuprofen. The study did not evaluate clinical semen parameters [5]. The aim of our study was to evaluate whether or not ibuprofen affects semen parameters.

## Materials and methods

**Fig. 1:**
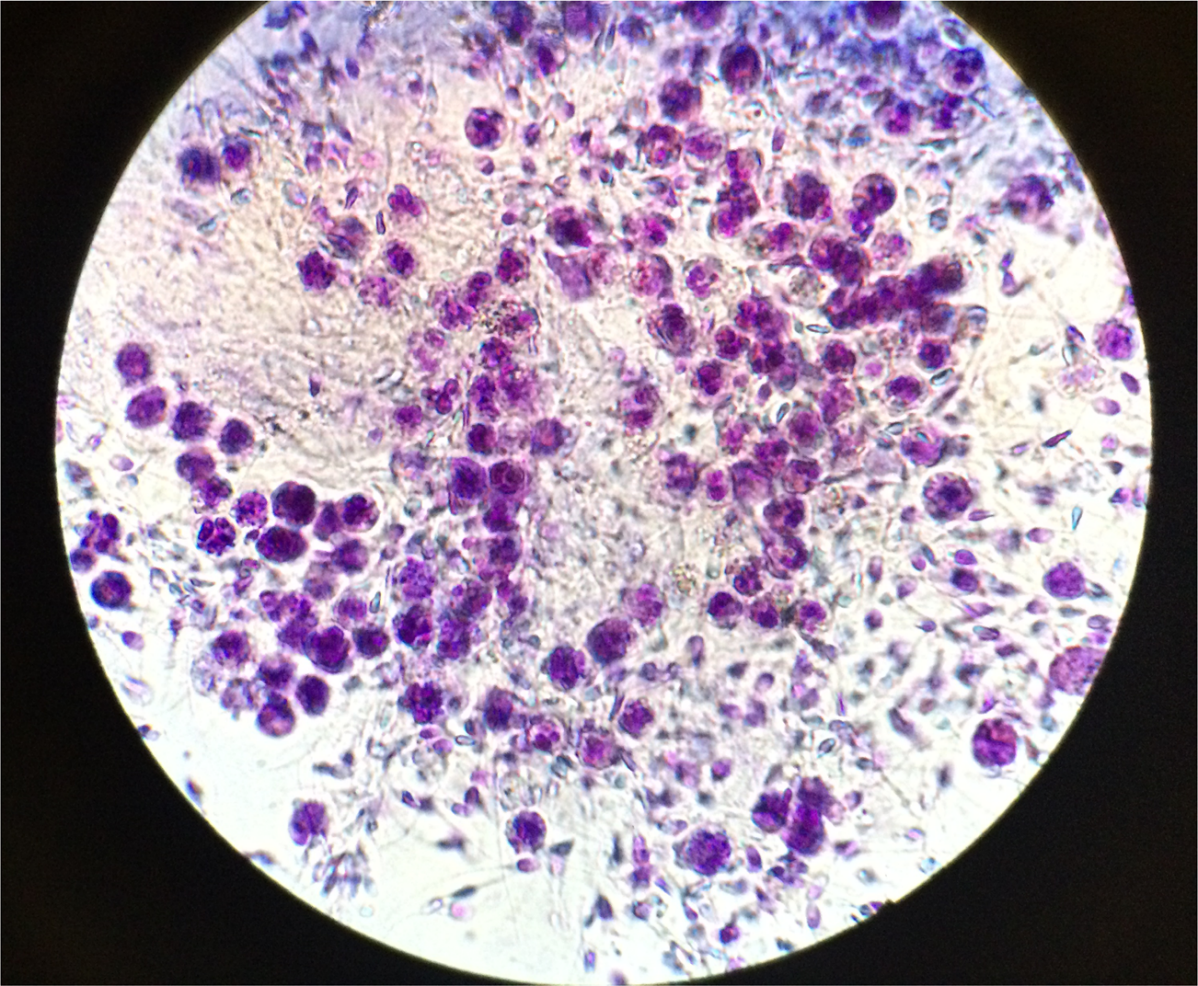
The microscopic appearance of seminal leukocytes after peroxidase staining in a semen sample consistent with leukocytospermia

Between October of 2012 and February of 2018, 64 men presenting for a fertility evaluation had semen analyses revealing leukocytospermia. Leukocytospermia was diagnosed by examining the semen of men with round cells in numbers greater than 5 million/ml in which peroxidase staining revealed seminal leukocytes in numbers greater than 1 million/ml (Fig. 1). The clinical treatment protocol for these men was a 3-week course of doxycycline 100 mg twice a day in combination with ibuprofen 600 mg every 8 hours (1800 mg per day) to treat potential infective and inflammatory processes regardless of semen culture results. Semen cultures were obtained on these patients and antibiotics were tailored if cultures grew organisms not sensitive to doxycycline. None of the men were treated with supplements or antioxidants for leukocytospermia. Repeated semen analyses were performed 3 weeks after initiating treatment to confirm that seminal leukocytes had resolved. Institutional Review Board exemption was obtained. Statistical analyses were performed via Student’s *t* test with a *p* value of < 0.05 considered statistically significant.

## Results

Out of the 64 men who were diagnosed with leukocytospermia and treated with doxycycline and ibuprofen, 51 of them returned for post-treatment semen analyses and were included in the data. Semen parameters evaluated for the purpose of this study included semen volume, sperm concentration, motility, total motile count (TMC), and sperm forward progressive motility. Morphology was excluded from the data as it could not be standardized between assessments with strict Kruger criteria versus WHO fourth edition criteria depending on the lab in which it was performed. Some patients had pre-treatment and post-treatment semen analyses at labs with differing criteria for the assessment of sperm morphology.

The mean age of the men included in the study was 35 (SD 4.6) years of age. There was not a statistically significant difference in mean abstinence intervals prior to semen analyses collected for the pre-treatment and post-treatment data. There was no statistically significant difference in semen volume, sperm concentration, motility, TMC, or forward progression after treatment with a 3-week course of ibuprofen at an 1800 mg dose per day (Table 1).

**Table 1:** Comparison of days of abstinence prior to collecting semen analysis sample as well as semen parameters in men pre-treatment and post-treatment with ibuprofen. Results are represented as means with standard deviations

	Days of abstinence	Volume (ml)	Concentration (mil/mL)	Motility (%)	TMC (millions)	Forward progression (%)
Pre	3.8 (1.4)	2.8 (1.2)	40.8 (39.9)	54.0 (21.1)	59 (80.2)	28.6 (3.9)
Post	3.8 (1.0)	2.7 (1.4)	39.1 (36.5)	54.6 (22.2)	69.6 (82.3)	28.0 (21.3)
*p* value	0.90	0.70	0.82	0.89	0.51	0.84

All 51 men with leukocytospermia had semen cultures performed, of which six (11.8%) grew out bacteria and the remainder revealed no growth of bacteria. All six samples with positive cultures grew out *Enterococcus* species. Of the 51 men included in the data, 4/51 (7.8%) smoked tobacco regularly, 34/51 (74.5%) consumed alcoholic beverages regularly with a mean number of alcoholic beverages per week of 4 (SD 5.2), and 2/51 (3.9%) of the men smoked marijuana regularly. Criteria for serum hormone evaluation included a sperm concentration of less than ten million/ml or symptoms raising suspicion for hypogonadism such as erectile dysfunction or low libido. Twenty-seven out of the 51 men (52.9%) met the criteria to undergo serum hormone evaluation which included total testosterone, FSH, luteinizing hormone (LH), prolactin, and estradiol. Of these 27 men, 10 (37%) were found to have hormone level abnormalities defined as those outside of laboratory reference ranges. Of the ten men with hormone abnormalities, one was found to have mild hyperprolactinemia with a prolactin of 24.7 ng/mL and a negative pituitary magnetic resonance imaging (MRI), one was found to have an elevated FSH of 25 IU/mL, and eight of whom were found to be hypogonadal with a mean serum total testosterone level of 211.9 ng/dL (SD 60.3).

## Discussion

There is concern that semen parameters among men are worsening over time and men overall are becoming less fertile [6–8]. It is crucial to identify the possible causes resulting in this decline. The impact of many environmental factors on semen parameters have been evaluated over the years to assist in counseling of subfertile men in efforts to optimize their semen parameters and overall fertility. Commonly used medications are not always assessed for their effect on male fertility prior to being released in the market. Ibuprofen is a commonly used over-the-counter analgesic in men of reproductive age, and such over-the-counter analgesics are among the most commonly used medications worldwide [9].

A recent study investigated the impact of ibuprofen on testicular physiology and concluded that ibuprofen can induce a state of compensated hypogonadism, which was defined as maintaining a normal serum testosterone level but having an elevation in LH. The clinical data was obtained in 31 men after treating 17 of these men with placebo and 14 of these men with ibuprofen. This data revealed that administration of ibuprofen to these men did not significantly impact FSH levels, total testosterone levels, free testosterone levels, sex hormone binding globulin (SHBG) levels, or estradiol levels. LH did rise in these men and a decrease in testosterone to LH ratio was described, which is primarily due to the rise in LH. Clinical semen parameters were not evaluated in this study. Assessment of ibuprofen’s impact on testosterone production, performed ex vivo and in vitro using testicular tissue as well as cell line in culture, revealed inhibition of Leydig cell production of testosterone which was dose-dependent with administration of ibuprofen. There were no significant changes in spermatogenic cells in these experiments [5].

A prior retrospective study of a total of 1082 men presenting for semen analysis, evaluated 68 of these men who took ibuprofen in the 3 months prior to their semen analysis. The authors found no statistically significant differences in semen parameters in those who took ibuprofen regularly compared to those who took ibuprofen intermittently, compared to those that did not use it at all [10].

Our current study allowed us to use the patients as their own controls to compare their semen parameters prior to and after ibuprofen use, and the study evaluates the clinically relevant semen parameters, excluding morphology with the inability to standardize for differing criteria for assessment of morphology at different labs. The objective of our study was not to assess hormonal parameters, only semen parameters in men presenting for fertility evaluations.

Limitations to this study include the retrospective nature with the inherent biases and the potential impact of doxycycline on semen parameters, which was prescribed for the same interval as ibuprofen was administered, prior to repeating semen analyses. However, semen parameters did not worsen or improve after treatment with doxycycline in this study, which is consistent with a previous publication revealing that doxycycline treatment in men with leukocytospermia had no significant impact on semen parameters [11]. The sample size was also a limitation; however, the number of men included in this study far exceeded the 31 men enrolled in the previous study, raising concern about the potential impact of ibuprofen on clinical testicular function [5]. A consideration was the time frame of repeating semen analyses following ibuprofen treatment. Although 3 weeks does not allow for a sufficient time for the assessment of improvement in semen parameters after a treatment, our goal was to demonstrate that there was not a detrimental impact on semen parameters, which a 3-week interval is adequate for to demonstrate. Transient insults to spermatogenesis from exposures and environmental factors typical occur very rapidly.

## Conclusions

Among men with leukocytospermia, the treatment that included a 3-week course of ibuprofen at 1800 mg per day did not demonstrate a statistically significant adverse impact on semen volume, sperm concentration, motility, TMC, or forward progressive motility when compared to pre-treatment semen analyses parameters. This suggests that ibuprofen in moderate doses for short intervals does not adversely impact male fertility clinically.
